# Supplementary material for: Novel Gold Nanorods@Thiolated Pectin on the Killing of HeLa Cells by Photothermal Ablation
Source: Pharmaceutics. 2023 Nov 2;15(11):2571. doi: 10.3390/pharmaceutics15112571 (PMC10675277; doi:10.3390/pharmaceutics15112571)
Supplement: Supplementary file 1 [file pharmaceutics-15-02571-s001.zip › pharmaceutics-2627210-supplementary.pdf]

Supplementary-Information

Novel Gold Nanorods@Thiolated Pectin on the Killing of HeLa Cells by Photothermal Ablation

Osvaldo Beltran [1], Mariangel Luna [1], Marisol Gastelum [1], Alba Costa-Santos [2,3], Adriana Cambón [2,3], Pablo Taboada [2,3], Marco A. López-Mata [4], Antonio Topete [5] and Josue Juarez [1,6,*]

1. DLS.

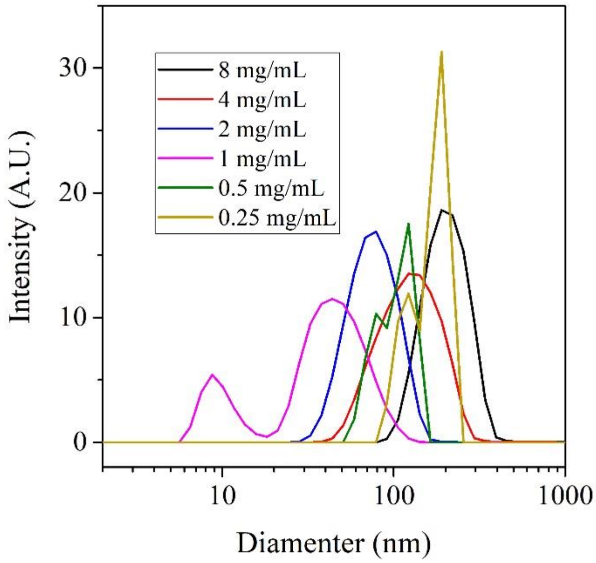

2. The setup for Zeta Potential Measurements

Sample: 0.25 mg/mL SH-PEC

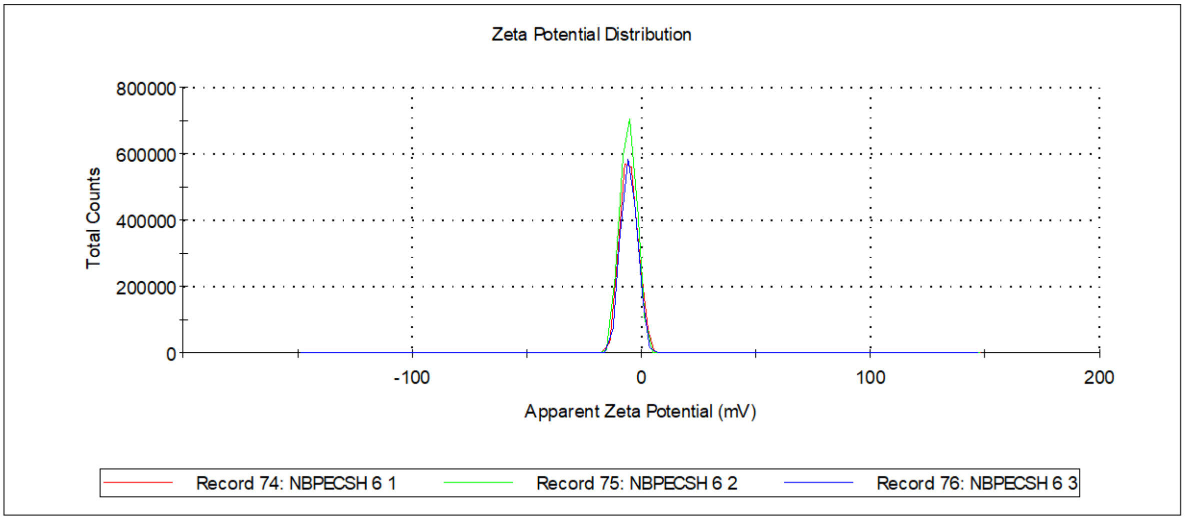

Sample: 0.5 mg/mL SH-PEC

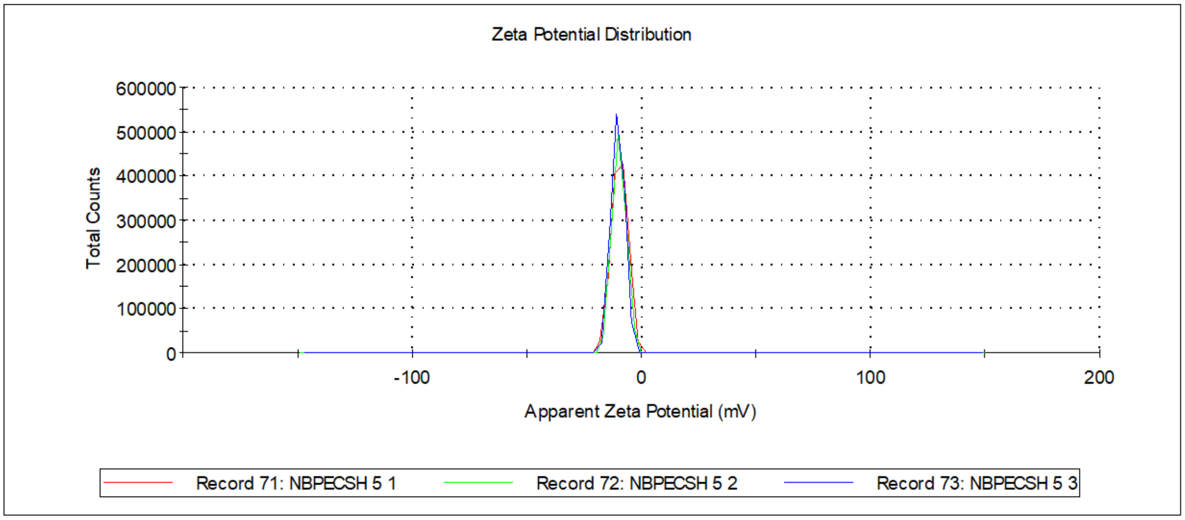

Sample: 1.0 mg/mL SH-PEC

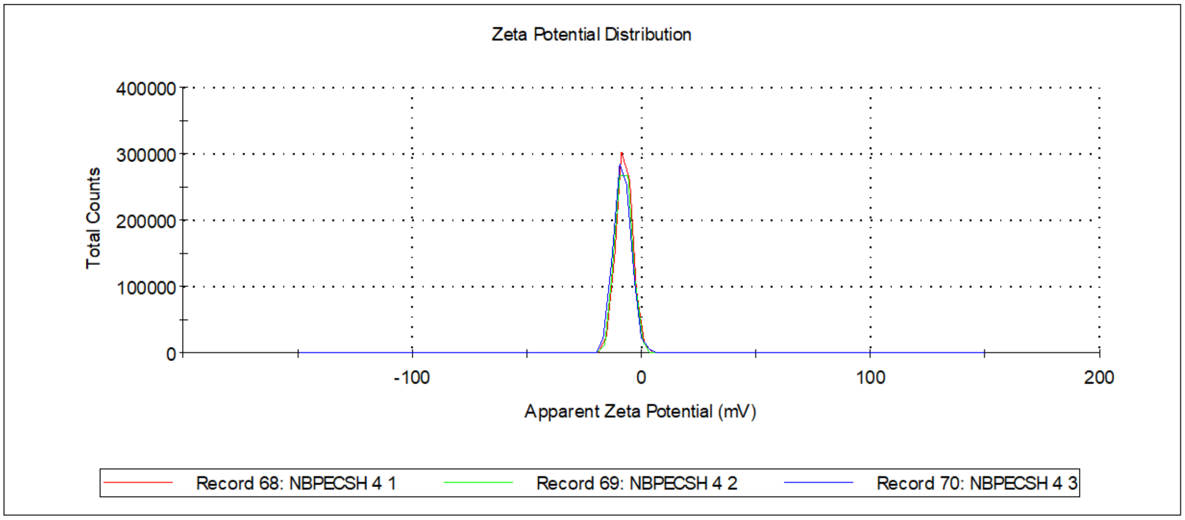

Sample: 2.0 mg/mL SH-PEC

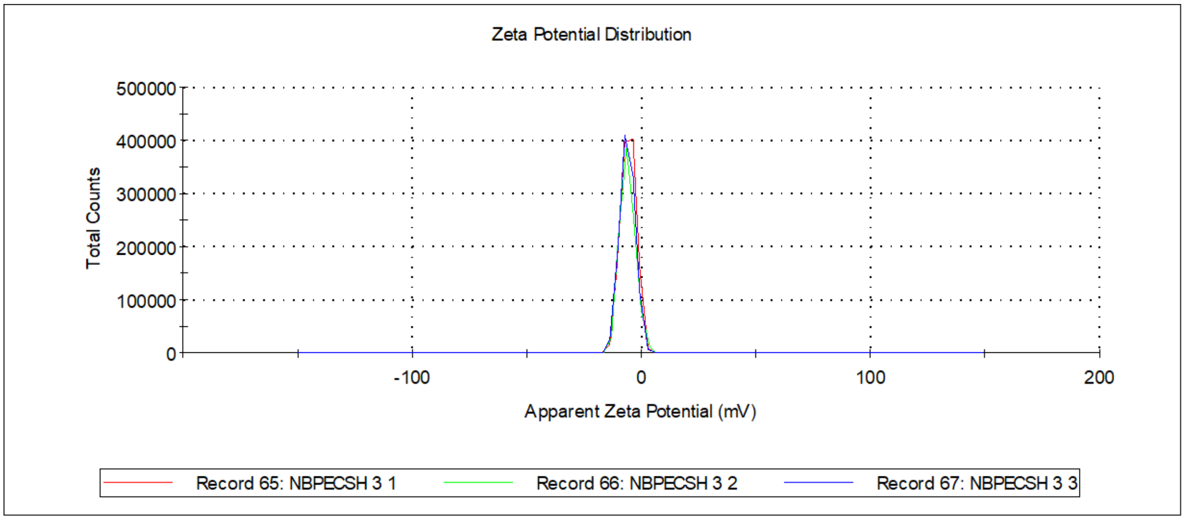

Sample: 4.0 mg/mL SH-PEC

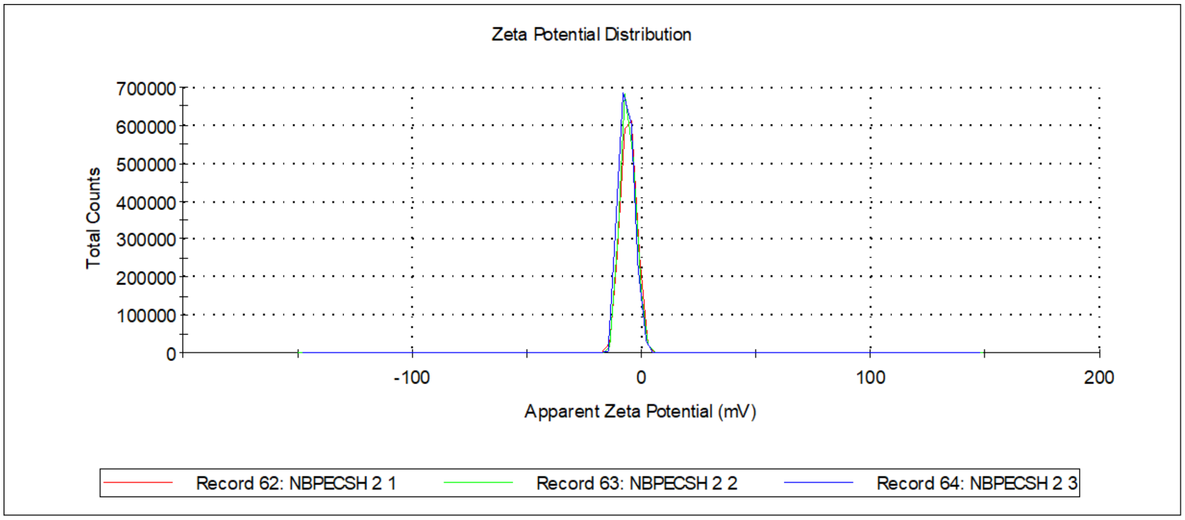

Sample: 8.0 mg/mL SH-PEC

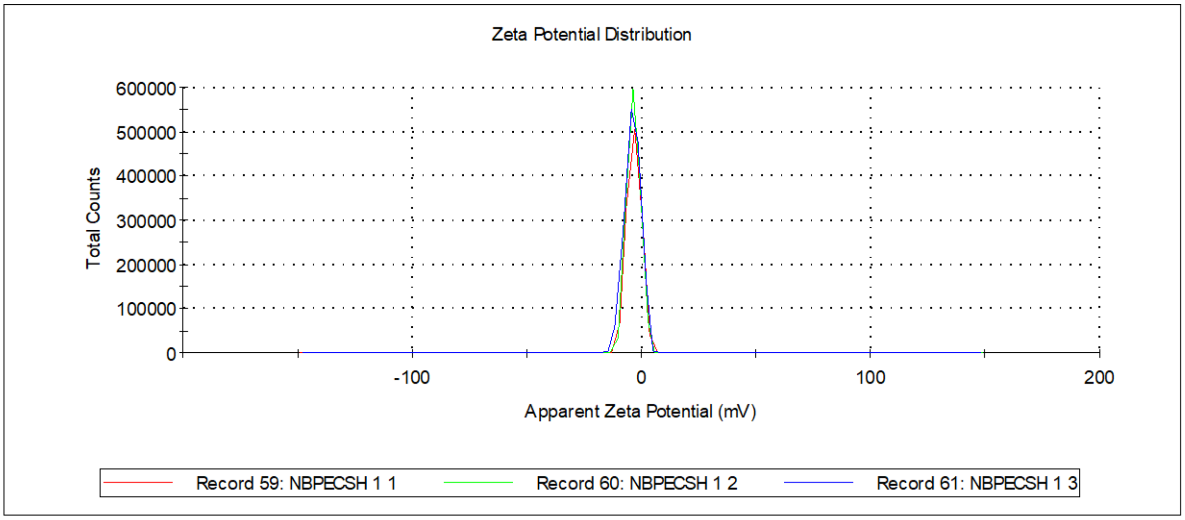

3.0 Photothermal assays

Temperature versus time recorded during the laser irradiation cycles (Figure 2 A, C, E, G, and I). These experimental data were adjusted to Roper equation ($\eta = \frac{hS(T_{eq}-T_{surr})-Q_0}{P(1-10^{-A_{808}})}$) to determine the photothermal conversion efficiency (η) of the AuNRs stabilized with different concentrations of SH-PEC.

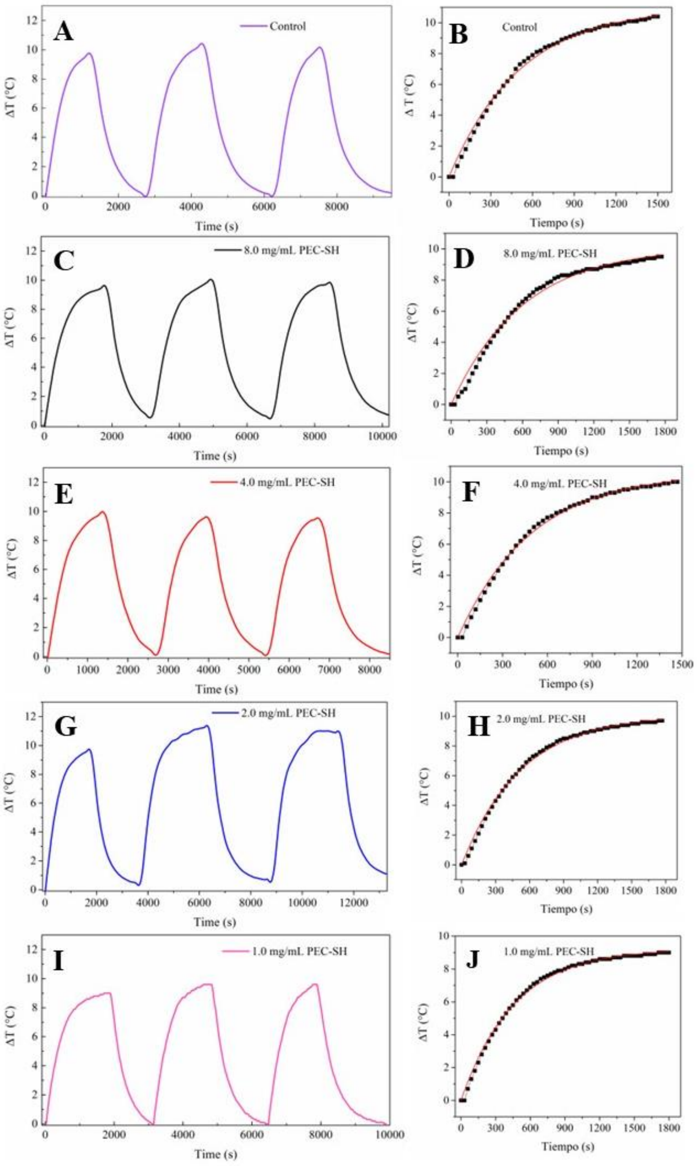

Roper, D. K., Ahn, W., & Hoepfner, M. (2007). *Microscale Heat Transfer Transduced by Surface Plasmon Resonant Gold Nanoparticles*. https://doi.org/10.1021/jp064341w

4.0 Statistical data.

T-Test

Viability Without Irradiation

Effect on [AuNRs] in cell viability of Balb/c 3t3 cell line

Column A	Control
vs	vs
Column B	1 mg/mL

Unpaired t test	
P value	0.0036
P value summary	**
Are means signif. different? (P < 0.05)	Yes
One- or two-tailed P value?	Two-tailed
t, df	t=6.118 df=4

Column A	Control
vs	vs
Column C	2 mg/mL

Unpaired t test	
P value	0.0063
P value summary	**
Are means signif. different? (P < 0.05)	Yes
One- or two-tailed P value?	Two-tailed
t, df	t=5.244 df=4

Column A	Control
vs	vs
Column D	4 mg/mL

Unpaired t test	
P value	0.0225
P value summary	*
Are means signif. different? (P < 0.05)	Yes
One- or two-tailed P value?	Two-tailed
t, df	t=3.612 df=4

Column A	Control
vs	vs

Column E	8 mg/mL

Unpaired t test	
P value	0.064
P value summary	ns
Are means signif. different? (P < 0.05)	No
One- or two-tailed P value?	Two-tailed
t, df	t=2.540 df=4

Column A	Control
vs	vs
Column B	1 mg/mL

Unpaired t test	
P value	< 0.0001
P value summary	***
Are means signif. different? (P < 0.05)	Yes
One- or two-tailed P value?	Two-tailed
t, df	t=26.88 df=4

Column A	Control
vs	vs
Column C	2 mg/mL

Unpaired t test	
P value	0.0021
P value summary	**
Are means signif. different? (P < 0.05)	Yes
One- or two-tailed P value?	Two-tailed
t, df	t=7.039 df=4

Column A	Control
vs	vs
Column D	4 mg/mL

Unpaired t test	
P value	< 0.0001
P value summary	***
Are means signif. different? (P < 0.05)	Yes
One- or two-tailed P value?	Two-tailed
t, df	t=20.00 df=4

Column A	Control
vs	vs
Column E	8 mg/mL

Unpaired t test	
P value	0.0006
P value summary	***
Are means signif. different? (P < 0.05)	Yes
One- or two-tailed P value?	Two-tailed
t, df	t=9.815 df=4

Column A	Control
vs	vs
Column B	1 mg/mL

Unpaired t test	
P value	< 0.0001
P value summary	***
Are means signif. different? (P < 0.05)	Yes
One- or two-tailed P value?	Two-tailed
t, df	t=28.91 df=4

Column A	Control
vs	vs
Column C	2 mg/mL

Unpaired t test	
P value	< 0.0001
P value summary	***
Are means signif. different? (P < 0.05)	Yes
One- or two-tailed P value?	Two-tailed
t, df	t=30.78 df=4

Column A	Control
vs	vs
Column D	4 mg/mL

Unpaired t test	
P value	0.0002
P value summary	***

Are means signif. different? (P < 0.05)	Yes
One- or two-tailed P value?	Two-tailed
t, df	t=13.20 df=4

Column A	Control
vs	vs
Column E	8 mg/mL

Unpaired t test	
P value	0.0011
P value summary	**
Are means signif. different? (P < 0.05)	Yes
One- or two-tailed P value?	Two-tailed
t, df	t=8.378 df=4

Column A	Control
vs	vs
Column B	1 mg/mL

Unpaired t test	
P value	< 0.0001
P value summary	***
Are means signif. different? (P < 0.05)	Yes
One- or two-tailed P value?	Two-tailed
t, df	t=15.79 df=4

Column A	Control
vs	vs
Column C	2 mg/mL

Unpaired t test	
P value	< 0.0001
P value summary	***
Are means signif. different? (P < 0.05)	Yes
One- or two-tailed P value?	Two-tailed
t, df	t=19.93 df=4

Column A	Control
vs	vs
Column D	4 mg/mL

Unpaired t test	
P value	< 0.0001
P value summary	***
Are means signif. different? (P < 0.05)	Yes
One- or two-tailed P value?	Two-tailed
t, df	t=41.37 df=4

Column A	Control
vs	vs
Column E	8 mg/mL

Unpaired t test	
P value	< 0.0001
P value summary	***
Are means signif. different? (P < 0.05)	Yes
One- or two-tailed P value?	Two-tailed
t, df	t=16.21 df=4

Column A	Control
vs	vs
Column B	1 mg/mL

Unpaired t test	
P value	0.0003
P value summary	***
Are means signif. different? (P < 0.05)	Yes
One- or two-tailed P value?	Two-tailed
t, df	t=12.21 df=4

Column A	Control
vs	vs
Column C	2 mg/mL

Unpaired t test	
P value	0.0003
P value summary	***
Are means signif. different? (P < 0.05)	Yes
One- or two-tailed P value?	Two-tailed
t, df	t=11.50 df=4

Column A	Control

vs	vs
Column D	4 mg/mL

Unpaired t test	
P value	0.0001
P value summary	***
Are means signif. different? (P < 0.05)	Yes
One- or two-tailed P value?	Two-tailed
t, df	t=15.18 df=4

Column A	Control
vs	vs
Column E	8 mg/mL

Unpaired t test	
P value	0.0003
P value summary	***
Are means signif. different? (P < 0.05)	Yes
One- or two-tailed P value?	Two-tailed
t, df	t=11.85 df=4

T-Test

Viability Without Irradiation

Effect on [AuNRs] in cell viability of HeLa cell line

Column A	Control
vs	vs
Column B	1 mg/mL

Unpaired t test	
P value	0.0024
P value summary	**
Are means signif. different? (P < 0.05)	Yes
One- or two-tailed P value?	Two-tailed
t, df	t=6.828 df=4
Table Analyzed	6250000000
Column A	Control
vs	vs
Column C	2 mg/mL
Unpaired t test	

P value	< 0.0001
P value summary	***
Are means signif. different? (P < 0.05)	Yes
One- or two-tailed P value?	Two-tailed
t, df	t=20.99 df=4
Table Analyzed	6250000000
Column A	Control
vs	vs
Column D	4 mg/mL
Unpaired t test	
P value	0.0006
P value summary	***
Are means signif. different? (P < 0.05)	Yes
One- or two-tailed P value?	Two-tailed
t, df	t=9.764 df=4
Table Analyzed	6250000000
Column A	Control
vs	vs
Column E	8 mg/mL
Unpaired t test	
P value	0.0019
P value summary	**
Are means signif. different? (P < 0.05)	Yes
One- or two-tailed P value?	Two-tailed
t, df	t=7.244 df=4
Table Analyzed	12500000000
Column A	Control
vs	vs
Column B	1 mg/mL
Unpaired t test	
P value	0.0011
P value summary	**
Are means signif. different? (P < 0.05)	Yes
One- or two-tailed P value?	Two-tailed
t, df	t=8.428 df=4
Table Analyzed	12500000000
Column A	Control
vs	vs
Column C	2 mg/mL

Unpaired t test	
P value	0.0006
P value summary	***
Are means signif. different? (P < 0.05)	Yes
One- or two-tailed P value?	Two-tailed
t, df	t=9.855 df=4
Table Analyzed	12500000000
Column A	Control
vs	vs
Column D	4 mg/mL

Unpaired t test	
P value	0.0002
P value summary	***
Are means signif. different? (P < 0.05)	Yes
One- or two-tailed P value?	Two-tailed
t, df	t=12.85 df=4
Table Analyzed	12500000000
Column A	Control
vs	vs
Column E	8 mg/mL

Unpaired t test	
P value	0.0002
P value summary	***
Are means signif. different? (P < 0.05)	Yes
One- or two-tailed P value?	Two-tailed
t, df	t=13.19 df=4
Table Analyzed	25000000000
Column A	Control
vs	vs
Column B	1 mg/mL

Unpaired t test	
P value	0.0108
P value summary	*
Are means signif. different? (P < 0.05)	Yes
One- or two-tailed P value?	Two-tailed
t, df	t=4.499 df=4
Table Analyzed	25000000000
Column A	Control
vs	vs
Column C	2 mg/mL

Unpaired t test	
P value	0.0029
P value summary	**
Are means signif. different? (P < 0.05)	Yes
One- or two-tailed P value?	Two-tailed
t, df	t=6.477 df=4
Table Analyzed	25000000000
Column A	Control
vs	vs
Column D	4 mg/mL
Unpaired t test	
P value	0.0002
P value summary	***
Are means signif. different? (P < 0.05)	Yes
One- or two-tailed P value?	Two-tailed
t, df	t=13.53 df=4
Table Analyzed	25000000000
Column A	Control
vs	vs
Column E	8 mg/mL
Unpaired t test	
P value	0.0021
P value summary	**
Are means signif. different? (P < 0.05)	Yes
One- or two-tailed P value?	Two-tailed
t, df	t=7.052 df=4
Table Analyzed	50000000000
Column A	Control
vs	vs
Column B	1 mg/mL
Unpaired t test	
P value	0.001
P value summary	***
Are means signif. different? (P < 0.05)	Yes
One- or two-tailed P value?	Two-tailed
t, df	t=8.674 df=4
Table Analyzed	50000000000
Column A	Control
vs	vs

Column C	2 mg/mL
Unpaired t test	
P value	0.0004
P value summary	***
Are means signif. different? (P < 0.05)	Yes
One- or two-tailed P value?	Two-tailed
t, df	t=10.66 df=4
Table Analyzed	50000000000
Column A	Control
vs	vs
Column D	4 mg/mL
Unpaired t test	
P value	0.0011
P value summary	**
Are means signif. different? (P < 0.05)	Yes
One- or two-tailed P value?	Two-tailed
t, df	t=8.383 df=4
Table Analyzed	50000000000
Column A	Control
vs	vs
Column E	8 mg/mL
Unpaired t test	
P value	0.0113
P value summary	*
Are means signif. different? (P < 0.05)	Yes
One- or two-tailed P value?	Two-tailed
t, df	t=4.448 df=4
Table Analyzed	1E+11
Column A	Control
vs	vs
Column B	1 mg/mL
Unpaired t test	
P value	< 0.0001
P value summary	***
Are means signif. different? (P < 0.05)	Yes
One- or two-tailed P value?	Two-tailed
t, df	t=37.10 df=4
Table Analyzed	1E+11
Column A	Control

vs	vs
Column C	2 mg/mL
Unpaired t test	
P value	0.0005
P value summary	***
Are means signif. different? (P < 0.05)	Yes
One- or two-tailed P value?	Two-tailed
t, df	t=10.48 df=4
Table Analyzed	1E+11
Column A	Control
vs	vs
Column D	4 mg/mL
Unpaired t test	
P value	0.0002
P value summary	***
Are means signif. different? (P < 0.05)	Yes
One- or two-tailed P value?	Two-tailed
t, df	t=12.67 df=4
Table Analyzed	1E+11
Column A	Control
vs	vs
Column E	8 mg/mL
Unpaired t test	
P value	0.0116
P value summary	*
Are means signif. different? (P < 0.05)	Yes
One- or two-tailed P value?	Two-tailed
t, df	t=4.409 df=4

T-Test

Viability With Irradiation

Effect on [AuNRs] in cell viability of Balb/c 3t3

Table Analyzed	Balb
Column A	Control
vs	vs
Column B	1 mg/mL

Unpaired t test	
P value	0.0017
P value summary	**
Are means signif. different? (P < 0.05)	Yes
One- or two-tailed P value?	Two-tailed
t, df	t=7.5 df=4
Table Analyzed	Balb
Column A	Control
vs	vs
Column C	2 mg/mL

Unpaired t test	
P value	0.0014
P value summary	**
Are means signif. different? (P < 0.05)	Yes
One- or two-tailed P value?	Two-tailed
t, df	t=7.939 df=4
Table Analyzed	Balb
Column A	Control
vs	vs
Column D	4 mg/mL

Unpaired t test	
P value	0.0016
P value summary	**
Are means signif. different? (P < 0.05)	Yes
One- or two-tailed P value?	Two-tailed
t, df	t=7.569 df=4
Table Analyzed	Balb
Column A	Control
vs	vs
Column E	8 mg/mL

Unpaired t test	
P value	0.0023
P value summary	**
Are means signif. different? (P < 0.05)	Yes
One- or two-tailed P value?	Two-tailed
t, df	t=6.916 df=4

T-Test

Viability With Irradiation

Effect on [AuNRs] in cell viability of HeLa

Table Analyzed	HeLa
Column A	Control
vs	vs
Column B	1 mg/mL
Unpaired t test	
P value	< 0.0001
P value summary	***
Are means signif. different? (P < 0.05)	Yes
One- or two-tailed P value?	Two-tailed
t, df	t=17 df=4
Table Analyzed	HeLa
Column A	Control
vs	vs
Column C	2 mg/mL
Unpaired t test	
P value	< 0.0001
P value summary	***
Are means signif. different? (P < 0.05)	Yes
One- or two-tailed P value?	Two-tailed
t, df	t=16.96 df=4
Table Analyzed	HeLa
Column A	Control
vs	vs
Column D	4 mg/mL
Unpaired t test	
P value	0.0002
P value summary	***
Are means signif. different? (P < 0.05)	Yes
One- or two-tailed P value?	Two-tailed
t, df	t=13.60 df=4
Table Analyzed	HeLa
Column A	Control
vs	vs
Column E	8 mg/mL
Unpaired t test	
P value	0.0007
P value summary	***

Are means signif. different? (P < 0.05)	Yes
One- or two-tailed P value?	Two-tailed
t, df	t=9.409 df=4

T-Test

ROS Generation

Effect on [SH-PEC] in ROS Generation after irradiation in Balb/c 3t3 Cell line

Table Analyzed	Balb
Column B	Control (-)
vs	vs
Column C	1 mg/mL
Unpaired t test	
P value	0.0168
P value summary	*
Are means signif. different? (P < 0.05)	Yes
One- or two-tailed P value?	Two-tailed
t, df	t=3.9 df=4
Table Analyzed	Balb
Column B	Control (-)
vs	vs
Column D	2 mg/mL
Unpaired t test	
P value	0.0584
P value summary	ns
Are means signif. different? (P < 0.05)	No
One- or two-tailed P value?	Two-tailed
t, df	t=2.6 df=4
Table Analyzed	Balb
Column B	Control (-)
vs	vs
Column E	4 mg/mL
Unpaired t test	
P value	0.0024
P value summary	**
Are means signif. different? (P < 0.05)	Yes
One- or two-tailed P value?	Two-tailed
t, df	t=6.8 df=4
Table Analyzed	Balb
Column B	Control (-)

vs	vs
Column F	8 mg/mL
Unpaired t test	
P value	0.0008
P value summary	***
Are means signif. different? (P < 0.05)	Yes
One- or two-tailed P value?	Two-tailed
t, df	t=9.0 df=4

T-Test

ROS Generation

Effect on [SH-PEC] in ROS Generation after irradiation in HeLa cell line

Table Analyzed	HeLa
Column B	Control (-)
vs	vs
Column C	1 mg/mL
Unpaired t test	
P value	0.3855
P value summary	ns
Are means signif. different? (P < 0.05)	No
One- or two-tailed P value?	Two-tailed
t, df	t=0.97 df=4
Table Analyzed	HeLa
Column B	Control (-)
vs	vs
Column D	2 mg/mL
Unpaired t test	
P value	0.0034
P value summary	**
Are means signif. different? (P < 0.05)	Yes
One- or two-tailed P value?	Two-tailed
t, df	t=6.2 df=4
Table Analyzed	HeLa
Column B	Control (-)
vs	vs
Column E	4 mg/mL

Unpaired t test	
P value	0.0727
P value summary	ns
Are means signif. different? (P < 0.05)	No
One- or two-tailed P value?	Two-tailed
t, df	t=2.4 df=4
Table Analyzed	HeLa
Column B	Control (-)
vs	vs
Column F	8 mg/mL
Unpaired t test	
P value	0.0014
P value summary	**
Are means signif. different? (P < 0.05)	Yes
One- or two-tailed P value?	Two-tailed
t, df	t=7.9 df=4
